# Investigating the effect of the Vula Mobile app on coordination of care and capacity building in district health services, Cape Town: Convergent mixed methods study

**DOI:** 10.4102/safp.v63i1.5251

**Published:** 2021-09-22

**Authors:** Patrick Gloster, Robert Mash, Steve Swartz

**Affiliations:** 1 Division of Family Medicine and Primary Care, Faculty of Medicine and Health Sciences, Stellenbosch University, Cape Town, South Africa; 2 Metro Health Services, Western Cape Provincial Government, Cape Town, South Africa

**Keywords:** primary care, primary healthcare, coordination of care, capacity building, health information technology

## Abstract

**Background:**

Coordinating care is a defining characteristic of high quality primary care. Currently, very little is known about coordination of care in South Africa’s primary care setting. The Vula Mobile app was introduced in 2018 to assist with referring patients from primary care facilities to the Eerste River District Hospital (ERDH) emergency centre. The aim of this study was to evaluate the use of the app and its effect on coordination of care and capacity building of staff.

**Methods:**

Convergent mixed methods were used with quantitative data collected from hospital records and the Vula Mobile database, and with qualitative data collected from health professionals in primary care and the district hospital.

**Results:**

Out of 13 321 patients seen in the emergency centre of the district hospital over the 6-month study period, only 1932 (14.5%) of the patients were referred with Vula. Most of these referrals were accepted (85.5%). Sometimes, advice was given to (35.0%) or additional information was requested (27.4%) from the referring doctor. There was little use of Vula in providing other feedback (0.6%). The introduction of the Vula app led to a decrease in the number of inappropriately referred patients (6.7% to 4.2%, *p* = 0.004). Doctors using the Vula app perceived that it improved care coordination and had the potential for useful feedback.

**Conclusion:**

Vula improved coordination of patients referred from primary care facilities in the Metro Health Services to the district hospital, but missed the opportunity to support continuing professional development and learning. Utilisation of the Vula app should be increased and its potential to provide feedback should be enhanced. Attention should be given to reducing the number of patients self-referred or referred without using the Vula app.

## Introduction

Coordinating care for patients is a defining characteristic of effective primary care,^1^ and contributes to higher quality of care and increased efficiency.^2^ However, patients and providers perceive many failures in coordination of care.^3^ Both specialists and primary care providers (PCP) report that they are not getting the information they need from each other to enable high quality care.^4,5^ Both patients’ outcomes and practitioners’ satisfaction are highly reliant on effective communication.^4^

Coordination of care is the deliberate integration or organisation of activities between two or more participants involved in a patient’s care, in order to facilitate the appropriate delivery of healthcare services.^6^ It is often achieved by the exchange of information amongst practitioners responsible for different aspects of care.^6^ Coordination can be sequential, when responsibility for care is handed over from one team to another, or parallel, when teams are working simultaneously alongside each other.^7^ It is often confused with continuity of care. Continuity of care refers to ongoing care with the same practitioner, or sometimes a small team, that extends beyond specific episodes of illness or disease.^8^ Transitional care is another term that refers to a set of actions designed to ensure the coordination of healthcare as patients transfer between different locations or different levels of care.^9^ Good continuity of primary care can contribute to better sequential coordination as more accurate and comprehensive information may be available. Likewise, good sequential coordination may enable continuity of primary care as feedback is received to guide ongoing care.^7^

Patients undergoing transitional care are at risk of medical errors, service duplication, inappropriate care, and critical elements of the care plan ‘falling through the cracks’.^9^ The transitional care process for emergency referrals has four components that determine how effectively it is done^9^:

- Communication between sending and receiving clinicians. This includes a common care plan, a summary of care provided by the sending institution, the patient’s goals and preferences (including advanced directives), an updated list of the problems, baseline physical and cognitive functional status, medications and allergies and contact information for the patient’s caregiver(s) and PCP.
- Preparation of the patient and caregiver for what to expect at the next site of care.
- Review of medication of all patients. This includes reviewing the chronic medication that the patient routinely takes and deciding whether it needs to continue during the current transfer and admission, the medication given to the patient by the PCP at the referring centre for the current admission and the medication given at the accepting hospital by the accepting healthcare provider once the patient has arrived.
- A follow-up plan for how the results of tests performed during the current admission can be checked at the patient’s primary care facility (PCF) and for follow-up appointments with the PCP.

Many strategies have been proposed to improve transfer of information and transitional care such as shared electronic medical records, web-based referrals, referral guidelines and virtual consultations.^10^

Ultimately, poorly executed transitional care may lead to poor clinical outcomes, dissatisfaction amongst patients and doctors and inappropriate use of hospital, emergency and post-acute services.^4,11^

Insufficient transfer of information in the referral process between referring and accepting doctors (in both directions) is a common phenomenon. Up to 70% of specialists globally, rated background information received from referring providers as fair to poor,^12,13,14^ and up to 50% of referring practitioners desired more feedback from the specialists.^15,16,17^ Disagreements with or misunderstandings about management plans also exist between referring practitioners and specialists in up to 26% of referrals worldwide.^10^ Inadequate transfer of information between practitioners ultimately forces families to become the source of information, which many families are not comfortable with.^11^

Patients themselves presume that there is good communication between practitioners, access to all available information and an agreed-upon care pathway.^18^ They are mostly unaware of the challenges and processes needed to coordinate care and infer that care is coordinated when no problems occur.^18^

Unfortunately, there is little South African data on care coordination in the primary care setting. One study conducted in the Western Cape scored the coordination of information as good.^19^ This, however, was looking more at the availability of stored patient records in primary care as a pre-requisite for coordination, rather than the transitional emergency care process. However, one can confidently assume that if a study was done in South Africa on the quality of transitional emergency care, it would be similar to international results, which show that coordination of care is poor.^9^

The Vula Mobile app (www.vulamobile.com) is a relatively new development in South Africa that allows for back and forth text communication as well as pictures between the referring practitioner at the PCF and the accepting doctor at the referral centre. The application was designed and developed by Mafami (Pty) Ltd (‘Mafami’) and made available in 2014. It has grown extensively since then and currently Vula has just over 14 200 healthcare users country wide, with about 10% being specialists and is extending into neighbouring countries.^20^

The current data available from Vula are for referrals to specialist departments at three large hospitals in the Western Cape.^21^ The hospitals include: Tygerberg Hospital, Worcester Hospital and Khayelitsha Hospital, all of which offer specialist services. In this context, Vula reduced the number of transferred patients as specialists often gave advice to the PCP on managing the patient further in primary care.^21^ This was most significant for gynaecology at Worcester Hospital, where 52% of the referrals were managed with advice only.^21^ Another option was to give an out-patient appointment, thereby eliminating the need for an immediate emergency transfer. This was also most significant in orthopaedics at Worcester Hospital where 34% of the cases were deferred for outpatient appointments, with initial management in primary care.^21^

Until 2018, Vula was only used by doctors at district and primary levels of care to refer patients to specialist departments at regional and tertiary hospitals. No studies have looked at the use of Vula within the district health services themselves, and specifically for transitions between primary care and district hospitals. Although one can clearly see how using Vula will potentially improve transitional care in the district health services; there is a need to formally evaluate this.

The aim of this study, therefore, was to evaluate the use and effect of the Vula Mobile app for patients referred to the Eerste River District Hospital (ERDH) emergency centre from surrounding PCFs. Specific objectives included: to evaluate the uptake and use of Vula by PCPs, the effect of Vula on coordination of care and on capacity building in primary care.

## Methods

### Study design

Convergent mixed methods were used to evaluate the effect of the Vula app on coordination of care and capacity building. The study design evaluated the effects of implementing Vula and not the effectiveness compared to other interventions, which would have required a different design. A logic model (Table 1) was used to guide the collection of quantitative data from patient records and the Vula database, and descriptive exploratory qualitative data from semi-structured interviews with health professionals.

**TABLE 1:** Logic model for the implementation of Vula.

Inputs	Activities	Expected outputs	Desired outcomes
Download Vula	Discuss with practitioners that they should now use Vula as the official mechanism and means of communication when referring patients to the ERDH EC.	Registration and use of Vula for emergency referrals to the ERDH EC.	Decrease in the proportion of inappropriate referrals.
-	Follow-up training and reinforcement or troubleshooting as necessary.	-	-

ERDG EC, Eerste River District Hospital emergency centre.

### Setting

Eerste River District Hospital had a catchment area of approximately 61.0 km^2^ with a population of approximately 420 000 people.^22^ The ERDH emergency centre (EC) received patients from PCFs run by the Western Cape Government (WCG) and City of Cape Town (COCT). Western Cape Government facilities included a 24-h community health centre (CHC) and two community day centres (CDC). City of Cape Town facilities were four smaller clinics. Several private general practitioners (GPs) also referred patients to ERDH. All COCT PCFs were nurse-driven services, and all the WCG facilities had permanent doctors as well as nurses. Western Cape Government facilities offered comprehensive primary care, whilst COCT clinics had more vertical services specifically for human immunodeficiency virus (HIV), tuberculosis (TB), family planning and child health. City of Cape Town clinics were supported by visiting doctors.

The ERDH EC had an average daily turnover of about 70 patients and monthly turnover of about 2100 patients. The EC consisted of three resuscitation beds, six high care beds, 10 spaces in the nebulisation room, an overnight ward consisting of 15 beds, an isolation room and a spill over passage that could hold up to 25 patients if needed. Often, the EC was filled to maximum capacity and even over capacity, requiring re-organisation to accommodate the load. The staff included up to six doctors at different levels from interns to specialist family physicians, three clinical nurse practitioners, five staff nurses and a unit manager. The EC was under immense pressure with a significant workload and high ratio of patients to healthcare workers.

The South African Triage Score (SATS) is a widely used scale to classify the urgency of patients in an EC from their reported symptoms and vital signs. A score of 0–2 is green (least urgent), 3–4 is yellow, 5–6 is orange, and 7 or more is red (most urgent). The SATS tries to assist in the more urgent patients being seen soonest in a busy EC. According to the SATS, patients that are triaged as red need to be seen immediately, orange within 10 min, yellow within an hour, green within 4-h and blue (dead on arrival) within 2-h.^23^ Patients triaged as green could often be treated equally well in primary care and therefore a reduction in inappropriate referrals from PCFs should translate into a reduction in the proportion of patients triaged as ‘green’.

The researcher was working at ERDH as a family medicine registrar, rotating through all the departments and performed overtime in the EC. The researcher did not work at any of the PCFs in the study, but had frequent interaction with the practitioners working there. The researcher knows the staff working at ERDH well and is acutely aware of the challenges faced at this facility. Doctors at the ERDH EC used Vula both to refer patients to Tygerberg Hospital and to accept patients from the surrounding PCFs and private GPs.

### Study populations

Three different study populations were identified: patients who used the EC before and after the implementation of Vula, patients who were referred using Vula and health professionals who used Vula to help with transfers. The before and after study population was intended to evaluate whether there was a reduction in the non-urgent (so called ‘green’) patients seen in the EC after the introduction of Vula. The patient referred with Vula were identified and data were used to describe the patients, the referrals, interactions between the EC and the referring doctor as well as the final disposition. The health professionals from the study population were interviewed to explore their experience of using Vula.

In order to compare referrals before and after the introduction of Vula, it was necessary to compare a minimum of 580 patients. A sample size calculation was made based on the need to detect a 5% change in triage colour amongst patients with 90% power and 5% type 1 error. The following periods were compared to account for seasonal variation:

- January–February 2018 and January–February 2019.
- April–May 2018 and April–May 2019.
- July–August 2017 and July–August 2019.

Vula was implemented in May 2018 and so July–August 2019 had to be compared with the same period in 2017. In each 2-month period, a 2-week period was randomly selected. All patients presenting to the EC during the selected 2-week period were included in the study.

Time was given for the implementation of Vula from May 2018 to December 2018. Data on the use of Vula were then obtained from the Vula database for all patients referred with Vula from the same 6-month period (January–February 2019, April–May 2019 and July–August 2019).

All permanent and community service doctors working in the EC during the first half of 2019 as well as family medicine registrars either working or doing overtime in the EC were invited to participate in a focus group interview (FGI). All doctors working at the WCG PCFs were also invited to attend separate FGIs at the three WCG facilities. In total, therefore, four FGIs were conducted. In addition, two nurses chosen by the clinic manager from each WCG PCF were interviewed, totalling six nurses. Individual interviews were also conducted with two GPs that referred patients to the ERDH EC using Vula. At the time of the study, they were the only two GPs using Vula. City of Cape Town facilities were not using Vula, and therefore were not included.

### Implementation of Vula

Vula was implemented in May 2018 and was used to make referrals instead of phoning the ERDH EC or sending patients without discussion. All PCFs that were part of the WCG were expected to use Vula to refer patients to the EC. Other health professionals, such as GPs, could use Vula if they choose to do so. Vula was available on both iOS (iPhone operating system) and Android devices, and was free to download. It did not require any formal training to use. Once registered, the healthcare provider obtained a profile and selected which hospitals they needed for referral. For referrals to the ERDH EC, Vula provided six types of information to the receiving doctor:

- Referring doctor details (name and cell number).
- Patient identification (name, folder number/ID number, age) and location.
- Clinical information (each referral department on Vula has a specific format with both compulsory and optional fields. The ERDH EC had vital signs, Glasgow Coma Scale (GCS) score, diagnosis, current management, reason for referral).
- Chat history (this is the real time discussion between referring and accepting practitioners recorded with times of each submitted message).
- Photographs, for example, electrocardiograms or radiographs.

**FIGURE 1:**
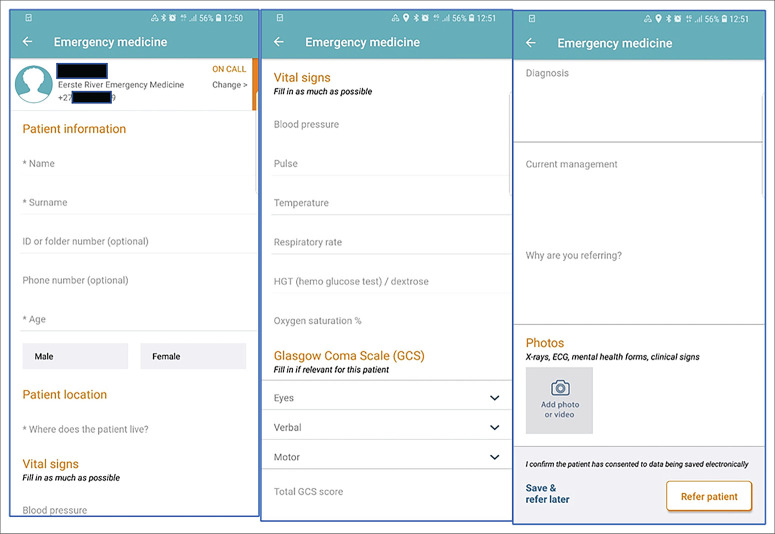
Screenshots of information provided on Vula.

Figure 1 shows screenshots of what the referring and receiving doctors saw on Vula.

Once the referral information was sent, the accepting doctor could review the available information and decide on a response. The ‘chat’ was then available for ongoing communication. Feedback could be given at any stage until the referral was closed by the receiving doctor. Table 1 shows the logic model for the intervention.

Vula provided opportunities to improve both coordination of care and formative feedback or capacity building to the PCP as shown in Table 2.

**TABLE 2:** Use of Vula to improve coordination and formative feedback.

Function	Referral	Arrival at EC	Care at EC	Outcome in EC
Care coordination	PCP sends referral information to the EC doctor. EC doctors can request additional information.	EC doctors can ask for additional information.	EC doctors can inform PCP of the progress.	EC doctors can inform the PCP of the outcome and any follow-up requirements.
Formative feedback or capacity building	EC doctors can provide feedback on appropriateness of referral or can provide advice on management (ongoing or prior to transfer).	EC doctors can inform PCP of patient’s condition on arrival and advise on how to improve initial care in future.	EC doctors can inform PCP on further assessment and management.	EC doctors can provide feedback on how to manage patients further on return to primary care.

PCP, primary care provider; EC, emergency centre.

### Data collection

Quantitative data were collected on the selected patients from the triage register in the EC on their triage colour (at the time of arrival) before and after the implementation of Vula.

Quantitative data were downloaded from the Vula database on patients referred with Vula. The total number of patients seen in the EC over the same period was also collected from the EC register. The following data were obtained from Vula:

- Number of referrals made.
- Reason for the referral.
- Patient’s primary care initial assessment or diagnosis.
- Response time by the accepting doctor.
- Amount and type of feedback or interaction.
- Outcome of referral, for example, accepted, not accepted, deferred elsewhere.

Semi-structured FGIs were conducted with the ERDH EC referring and accepting doctors to explore the effects of Vula on the referral process and changes in the amount and quality of feedback from before to after its implementation. The interviews were all conducted in the facilities in which the doctors were working. Individual semi-structured interviews were held with the GPs in their practices and the nurses were interviewed as dyads at the facilities where they worked. Interviews were conducted by the principal researcher in English or Afrikaans. The interviews were facilitated with the help of interview guides that provided an opening question and identified further topics to be explored with additional open questions to enable discussion of these topics. Topics covered included personal experiences using Vula, comparing Vula to the telephone as a referral mechanism and the amount and quality of feedback, either about the patient’s condition or as formative feedback. Interviews were audio recorded. Basic demographic data were collected on each participant to describe their characteristics in the results of the study.

### Data analysis

#### Quantitative data

Quantitative data were captured in Excel spread sheets and manually checked for any errors or omissions. The Statistical Package for the Social Sciences (SPSS) version 25 was used to analyse the data by the researcher, under supervision. Analysis used both descriptive and inferential statistics. Categorical data were analysed as frequencies and percentages, whilst numerical data were analysed as means and standard deviations (s.d.) or medians and interquartile ranges (IQRs), depending on how the data were distributed. The proportions of patients with different triage colours, from before to after the implementation of the Vula app, were compared using a Chi Square test.

#### Qualitative data

Audio recordings were transcribed verbatim and checked for errors or omissions against the recordings. Atlas-ti software was used to help with the analysis according to the framework method:^24^

- Familiarisation – The researcher familiarised himself with the transcripts and identified key issues and ideas emerging from the data that could be coded.
- Develop a coding index – The researcher identified and defined codes to be used and organised them into categories.
- Indexing – The researcher applied codes systematically to all qualitative data.
- Charting – The researcher re-arranged data into a series of charts, bringing all data with the same code and in the same category together.
- Interpretation – Each chart was read and the data interpreted in order to identify the key themes. The range and nature of ideas and experiences in each theme were described and interpreted.

#### Integration of data analyses

The quantitative and qualitative findings are presented in separate sections of the results and together provide complementary information and a fuller picture of how Vula impacted on coordination of care and capacity building. The qualitative findings shed light on how health professionals experienced the use of Vula and this helps to make sense of the quantitative findings on how the profile of patients changed in the EC and how Vula was used to make referrals and to enable interaction.

#### Trustworthiness of qualitative analysis

Analysis was supervised by R.M., particularly the construction of the coding index and the interpretation of findings. The analysis followed a clear stepwise process that could be audited with the help of Atlas-ti. Data from different respondents (PCPs vs. hospital doctors) were triangulated to provide a more in-depth understanding from both perspectives. The limited data from private GPs and nurses added some further viewpoints to expand the understanding of how Vula was used in other settings. The study setting and participants are described in as much detail as possible to enable readers to make decisions on the transferability of the findings. The researcher was mindful of his own reactions during the interviews and the data analysis to remain conscious of his own assumptions and beliefs that might influence the interpretation.

## Results

### Quantitative results

**FIGURE 2:**
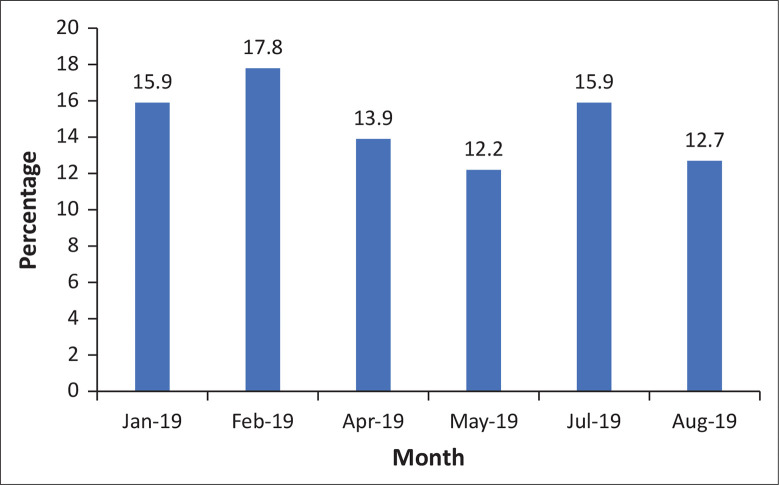
Percentage of patients at the Eerste River District Hospital emergency centre referred with the Vula Mobile app.

Overall, there were 13 321 patients seen in the ERDH EC over the 6-month period in 2019, of which 1932 (14.5%) were referred using the Vula Mobile app. Figure 2 shows the percentage of patients who were referred with Vula month by month. As all WCG facilities were using Vula (Table 3), this implies that only 12.5% of referrals came from the main public sector primary care platform. Only a few private GPs were using Vula (2.0% of all referrals).

**TABLE 3:** Characteristics of patients referred with the Vula Mobile app (*N* = 1932).

Characteristics	*n*	%
**Gender**		
Male	918	47.5
Female	1014	52.5
**Referring centre**		
Delft CHC	1298	67.2
Kleinvlei CDC	294	15.2
Symphony CDC	135	7.0
Private GPs	38	2.0
Other/unknown	167	8.6
**Reason for referral**		
Transfer to hospital	1880	97.3
Advice	47	2.4
Other/unknown	5	0.3
**Top 5 systems**		
Respiratory	372	19.4
Psychological	308	16.1
Gastrointestinal	223	11.7
General/unspecified	196	10.2
Cardiovascular	183	9.6
**Top 10 diagnoses**		
Psychosis	117	6.1
Pneumonia	107	5.5
Anaemia	93	4.8
Seizure/convulsion	85	4.4
Delirium	75	3.8
Skin infection	70	3.6
Diabetes insulin dependant	64	3.3
Heart failure	58	3.0
Abscess/carbuncle	49	2.5
Suicide attempt	48	2.5

CHC, community health centre; CDC, community day centre; GP, general practitioner.

**FIGURE 3:**
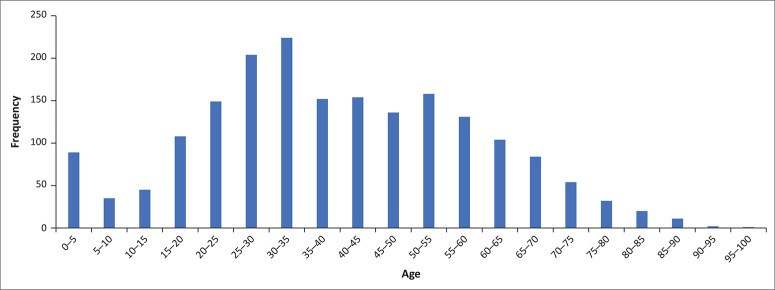
Age distribution of patients referred to the Eerste River District Hospital emergency centre with the Vula Mobile app.

Table 3 presents the characteristics of the patients referred with Vula. The patients were mostly young adults (mean age: 39.2 years, s.d: 19.3), and the age distribution of the patients referred is shown in Figure 3. There were slightly more females (52.5%) than males (47.5%). Most of the patients were referred from Delft CHC (67.2%), which is the largest 24-h facility. Almost all referrals on Vula were to request patient transfer (97.3%) and only a small number were to ask for advice. Psychological illnesses were prominent in both the ‘Top 5 systems’ and ‘Top 10 diagnoses’. This may be because of mental healthcare users (MHCU) made appropriate use of their PCFs and therefore were referred over Vula instead of self-presenting or being referred without discussion. This is encouraged by the ERDH EC as the mental healthcare act (MHCA) forms can first be checked by the EC doctor receiving the referral and any necessary corrections can be made before the patient is accepted and transferred across.

Table 4 records the interaction between the referring and the receiving doctor. Eight categories were analysed and scored by the researcher as either having had an interaction or not. An interaction was defined as a discrete series of messages within one of these categories. The commonest reason for an interaction was for the receiving doctor to give advice on further management in primary care or to request additional information on the referral. The median waiting time for a reply after the referral was sent was 16 min (IQR: 6–38). Although there was initially a fair amount of interaction, this stopped after the patient arrived at the ERDH EC.

**TABLE 4:** Summary of interactions over the Vula mobile app (*N* = 1868).

Interaction between referring and receiving doctor	*n*	%
EC doctors request additional information from referring doctors	512	27.4
EC doctors give feedback about the appropriateness of the referral	130	7.0
EC doctors give advice on further management whilst awaiting transfer	653	35.0
EC doctors request further information when patients arrive	0	0.0
EC doctors give feedback on patient’s condition or care received	2	0.1
EC doctors provide feedback on diagnosis/assessment	3	0.2
EC doctors provide feedback on disposal	3	0.2
EC doctors provide feedback on further management in primary care	1	0.1

EC, emergency centre.

**FIGURE 4:**
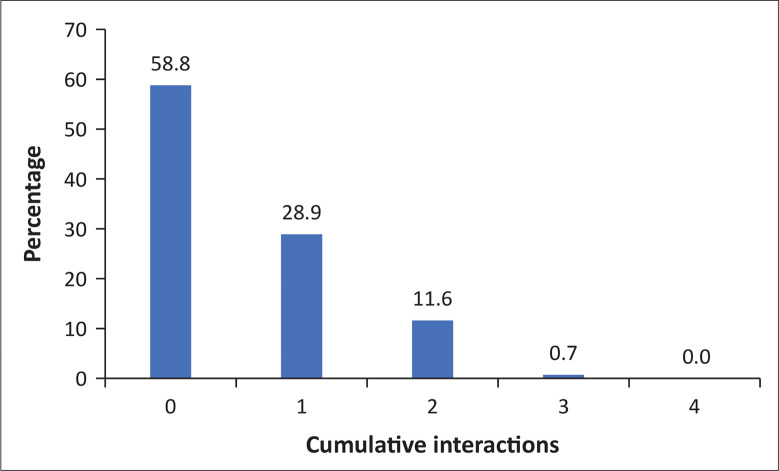
Cumulative interactions between referring and receiving doctors per patient (*N* = 1618).

Figure 4 demonstrates the cumulative interactions between referring and accepting doctors for patients who arrived at the EC. The majority (58.8%) had no interaction and very few had more than one interaction (12.3%).

Table 5 summarises the outcomes for the patients referred with the Vula app. Most of the referrals to the ERDH EC were accepted (85.5%). The referrals that were not accepted were all accompanied by feedback (2.6%) or were deferred for a more appropriate level of care (9.1%).

**TABLE 5:** Referral outcome for patients referred with the Vula Mobile app (*N* = 1868).

Referral outcome	*n*	%
Accepted with discussion	696	37.3
Accepted without discussion	901	48.2
Not accepted with discussion	48	2.6
Not accepted without discussion	0	0.0
Deferred for PHC or OPD follow-up	74	4.0
Deferred for higher level of care	96	5.1
No result	53	2.8

PHC, primary health care; OPD, outpatient department.

Table 6 shows the proportion of patients of different triage categories from before to after the implementation of Vula. There is a statistically significant decrease in the number of ‘green’ patients and an increase in ‘orange’ patients.

**TABLE 6:** Patient triage categories before and after the Vula Mobile app implementation.

Triage category	Pre Vula (*N* = 1229)		Post Vula (*N* = 1396)		*p*
	*n*	%	*n*	%	
Green	82	6.7	58	4.2	0.004
Yellow	607	49.4	660	47.3	0.280
Orange	484	39.4	629	45.1	0.003
Red	54	4.4	48	3.4	0.206
Blue	2	0.2	1	0.1	0.491

### Qualitative findings

All the doctors interviewed are described in Table 7, and all were using the Vula Mobile app. In addition, six nurse practitioners who were not using Vula were briefly interviewed, two from each health centre.

**TABLE 7:** Doctors taking part in interviews.

Facility	Number of participants	Male	Female	Temporary, for example, community service, registrar, locum	Permanent
Delft CHC	8	1	7	2	6
Kleinvlei CDC	5	0	5	2	3
Symphony Way CDC	2	1	1	1	1
Eerste River District Hospital	7	2	5	4	3
General practitioner	1	1	0	0	1
General practitioner	1	1	0	0	1

**Total**	**24**	**6**	**18**	**9**	**15**

CHC, community health centre; CDC, community day centre.

The findings are presented according to the following major themes:

- How Vula influenced the referral process?
- How Vula influenced the feedback given to referring doctors?
- How Vula influenced coordination of care between facilities?
- Why nurse practitioners were not using Vula to refer patients?

#### The influence of Vula on the referral process

Using Vula to refer patients was generally a good experience, with a quick response time and a better system than using the telephone. The process was efficient and whilst awaiting a response, the PCP could continue with other tasks. There were, however, times when responses were delayed and the process took too long. This was especially problematic for GPs, who preferred to completely sort out their patients during the consultation and did not want to ask them to wait, whilst they consulted the next patient:

‘With Vula I can literally refer the patient and go on doing whatever I need to do and wait on the response from the person on the other side.’ (Primary care provider, Symphony Way CDC)

The chat function allowed for ongoing discussion between referring and receiving doctors. Although this could be helpful, at times the referring doctors felt that this was causing unnecessary delays. It appeared that the receiving doctors tried to gather information relevant to the case, but not relevant to whether the patient required transfer:

‘… [*B*]ut sometimes I feel that they ask unnecessary questions, which is not going to change whether they’re going to accept the patient or not.’ (Primary care provider, Delft CHC)

Doctors who received the referrals could advise on the initial management of patients prior to transfer or defer the patient to a more appropriate setting, which was particularly helpful when receiving referrals from junior or new staff. The compulsory fields on Vula ensured that more complete, standardised and objective information was given. Pictures also frequently accompanied the referral. This benefitted the process by allowing the receiving doctor to advise on the initial management of the case, assist in the interpretation of side room investigations (e.g. electrocardiograms or radiographs) or make corrections to medico-legal documents (e.g. forms for certification of patients using the MHCA):

‘She realised that it wasn’t an appropriate referral for our level of care. So I think for her that was, I could see the response, she actually thanked me and said in the future she will know if she has a patient like this to refer onto a different level of care, or to make her do this and this instead of what she did.’ (Medical officer, Eerste River Hospital)

Patients were still transferred with a written referral note despite having the referral accepted electronically on Vula, which was seen as an unnecessary duplication of work.

Both the on call and referring doctor’s details were readily available on Vula. This assisted in knowing who one was discussing the patients with, as well as allowing alternative means of contact. Sometimes, it was necessary to call the accepting or referring doctor; and having the cell phone details meant that there was no struggle in getting hold of the person. Occasionally, phoning the on-call doctor directly was preferable, such as when referring multiple or critically ill patients; in such cases, it was helpful having the direct contact number of the on-call person. It was also easier to contact the same doctor again to follow up and find out what happened to the patient:

‘[What] Vula does also help with is it then shows who you can call. So it has their cell phone numbers.’ (Primary care provider, Kleinvlei CDC)

Referring and receiving doctors became familiar with each other and could personalise the referral by knowing the particular doctor’s preferences. Vula assisted with the accurate transfer of information by eliminating errors in telephonic communication because of, for example, strong accents or poor phone connectivity. It also eliminated tone from the conversation, which some referring doctors felt could adversely affect the referral process:

‘[*With*] Vula you don’t have to face the emotional problem. Because let me tell you, when I phone somebody and they say “Yes, what is it?” [*abrupt tone of voice*], already it makes me [*demonstrates confrontational body language*] if you understand, whereas with Vula you don’t have that.’ (Primary care provider, Delft CHC)

Accepting doctors from the ERDH EC enjoyed being on call with Vula, despite it sometimes feeling overwhelming. They felt more in control of the referrals and knew what to expect:

‘You get like four Vulas [*patients referred via Vula*]. It also helps you to know before and like what are you expecting, like this patient is really sick, I need to be ready when this patient comes.’ (Medical officer, Eerste River Hospital)

Accepting doctors also found it to be a good learning experience as they had to ensure that their advice was sound and they frequently discussed cases with other more experienced doctors within the ERDH EC if they were unsure. They could also defer patients to other facilities or to an outpatient appointment, thereby minimising the number of inappropriate patients ending up at the ERDH EC.

All doctors, whether referring or accepting patients, felt that there was more accountability because the referral and interaction were recorded on Vula. This ensured polite conversation both ways and eliminated the possibility of one party denying what was said or discussed:

‘It definitely creates a sense of accountability and responsibility for the person that is being referred to.’ (Primary care provider, Delft CHC)

Unfortunately, using a tech-based referral process meant relying on functioning infrastructure, that is electricity and cell phone reception, which was not always guaranteed. This was sometimes problematic for users. The cost of using one’s own data was also a potential problem:

‘If there’s not electricity there’s no connection because like you see I work with LTE [*Long-Term Evolution*] or wi-fi.’ (General practitioner, private practice)

Patients were mostly unaware that the referring and receiving doctors were using Vula on their cell phones and were unhappy that the doctors were constantly on their phones:

‘The patients also now start saying “No, the doctor is always on their phone!.”’ (Primary care practitioner, Kleinvlei CDC)

#### The influence of Vula on the feedback given to referring doctors

The referral process using Vula had a lot of potential for providing feedback. Feedback could be on the initial referral, management prior to transfer, assessment at the EC or final disposition. Despite this, little feedback was given to referring doctors, apart from at the time of the initial referral, where advice was sometimes given. The advice given was beneficial to both the referring doctor, who was still managing the patient, and the receiving doctor, who should have ended up caring for the same patient later that day and could ensure that definitive treatment started earlier:

‘We had a conversation about everything and discussed what else we could do for her here at Delft before sending her to you guys, which was really good.’ (Primary care provider, Delft CHC)

Feedback on initial management included advice on clinical care, deferral to more appropriate levels of care and correcting forms for the transfer of certified MHCU patients. It was widely agreed that the greater the knowledge gap between providers, the greater the benefit of the feedback. At times, Vula was only used by the PCP to ask for advice from a more senior hospital doctor and not for referral:

‘It’s a channel of communication and you know who’s on call for the day and then sometimes I just ask for advice and if they think that it needs to be referred or if they’re happy for us to manage it here and they can always re-discuss at a different day.’ (Primary care provider, Kleinvlei CDC)

After the transfer was arranged, however, there was almost no feedback. This was despite the accepting doctors wanting to give feedback and the referring doctors wanting to receive feedback. Referring doctors wanted feedback for their own personal development, to improve future referrals and patient care, and because they had an interest in the patients that they referred. A number of factors prevented further feedback from happening, such as patients being received at the hospital in a different shift because of the long waiting time for an ambulance, the EC being so busy that there was no time to give feedback or the patient was seen by a different doctor to the one who accepted them. Doctors who received referrals agreed that Vula would be a great platform to give feedback to the referring doctors, it was just not prioritised:

‘No, there’s no feedback.’ (Primary care provider, Delft CHC)

‘That [*feedback*] can actually help with kind of development of our skills.’ (Primary care provider, Kleinvlei CDC)

‘I think the lines are open and the potential is there.’ (Medical officer, Eerste River Hospital)

‘I definitely want some feedback.’ (General Practitioner, private practice)

#### The influence of Vula on coordination of care between facilities

Vula allowed for improved coordination of care. There was more trust built between practitioners at the different levels of care and better relationships developed than when using telephones. The direct nature of the conversations on Vula, which follows a similar format to other direct messaging applications, led to a friendlier interaction and a smoother referral process. Doctors from both referring and accepting facilities could educate the other about the resources available and gain insight into the other practitioner’s environment.

‘I think that once you get to know somebody you kind of get used to their style of referral and kind of the way that they treat patients and it kind of builds trust in a way, that, like you can’t let them, okay they can handle this or they’ve done what they said, they’re not kind of hiding things. It helps a lot.’ (Medical officer, Eerste River Hospital)

During the referral process, the accepting doctor could access patient information through other forms of technology to check results, medications or recent discharge summaries which were not always available to the referring doctors. This could be used to re-discuss with the referring doctor and potentially adjust the management or just prepare the accepting doctor for what was to come. During long delays in transfer, Vula also allowed the referring doctor to easily send updates of the patient’s condition to the accepting doctor, who could again give any additional advice or just take note of the evolving situation and be prepared:

‘So then it might be beneficial, you know, for them also to know what the condition of the patient is.’ (Primary care provider, Kleinvlei CDC)

When patients arrived at the ERDH EC, having the referral stored on Vula was very helpful. The clinical information allowed for a timeline of what the condition was at the time of the referral compared to the current condition. Pictures of electrocardiographs and radiographs were particularly beneficial for this comparison. It was also possible to easily trace the referring doctor if any more information was needed. The information on Vula was sometimes used to fill in the blanks if the patient was unable to answer the questions or if collateral history was no longer available:

‘I think what’s really great about Vula is because it’s this open chat that you’ve still got access to. Sometimes you can go back and ask stuff retrospectively.’ (Medical officer, Eerste River Hospital)

‘Even on ward rounds often you see an X-ray and you don’t know what the previous one looked like and we don’t have it, so then we can open it up on our phone and compared it, or look what the first ECG [*electrocardiogram*] looked like, that helps a lot.’ (Medical officer, Eerste River Hospital)

‘On ward rounds, sometimes the history that the MO this side got because of a patient being unaccompanied is you know, has got quite a lot of holes in. Then you like but that sounds familiar open up the referral but this is actually why they sent this patient, this is what they were concerned about because they often come here with you know a letter that may be not so detailed, or the letter’s lost and then we’re like “the patient is confused.”’ (Medical officer, Eerste River Hospital)

Discussions on Vula, sometimes, included higher levels of care when the referring doctors felt that the patient required regional or tertiary levels of care. If, however, the doctors at the higher level felt that the patient should come to the ERDH EC first, then the name of that doctor with whom the patient was discussed could easily be stored on Vula. If the patient then later required a transfer to the higher level of care, there was a name and number of the doctor at the higher centre with whom the case was earlier discussed:

‘I think that’s a good conversation, especially having the referring doctor from Tygerberg’s name just to say that we have discussed it [*when re-discussing a case that was deferred to the ERH EC from Tygerberg Hospital*].’ (Primary care practitioner, Delft CHC)

#### Why nursing practitioners are not using Vula to refer patients

Nurse practitioners were not using Vula to refer patients. They felt that Vula was only for the doctors, and that they had not received a formal invite to use it or had not received a formal invite or training to use it. The nurses working in facilities that had doctors felt that they would rather ask the doctors on site and would be more comfortable with the doctors referring the patents to the district hospital. However, they felt that it would be beneficial for their learning and they would have been willing to try to use it. They were also reluctant to use their personal data for Vula:

‘I thought it was not for nurses.’ (CNP, Kleinvlei CDC)

‘I wouldn’t have a problem using it.’ (CNP, Symphony Way CDC)

‘I also had to phone, it was long on the phone, hanging on, the reception was not answering for the doctor on call, in such a case Vula would have been really helpful.’ (CNP, Symphony Way CDC)

## Discussion

**FIGURE 5:**
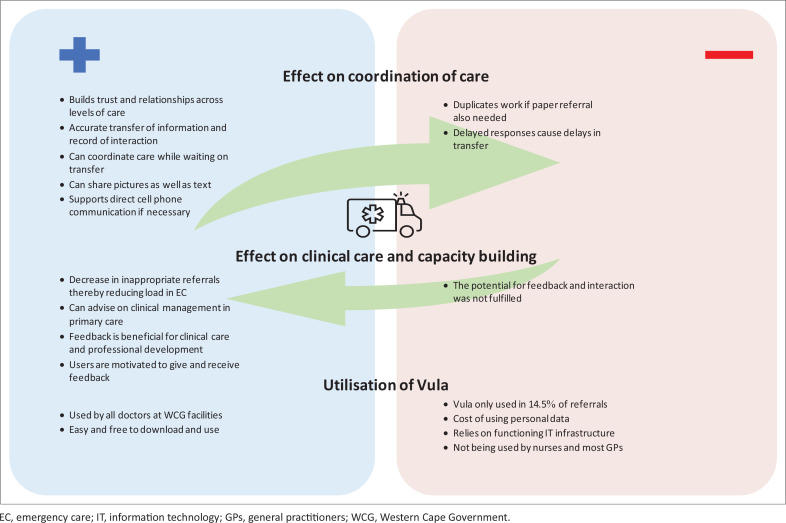
Summary of the key findings.

Figure 5 presents the key findings of the study in a conceptual framework. The key findings have been organised into three main categories showing how the Vula Mobile app has an effect on the coordination of care, clinical care and capacity building, as well as utilisation of the Vula app. The key findings are summarised in terms of their positive and negative aspects under these three categories.

Patients in South Africa often inappropriately seek healthcare at their district hospital EC instead of their PCF, especially after hours.^25,26,27^ Almost all referrals from WCG facilities and some referrals from GPs were made with Vula, but these only represented 14.5% of all patients seen in the EC. The only time the PCPs, working in the WCG facilities, did not use Vula, was when they were locum doctors who had not yet gained access to Vula. Private GPs mostly refer to private facilities unless the patient cannot afford this. Most of patients in the EC were, therefore, self-referred. Vula can be used as a tool to decrease the number of inappropriate patients being seen at the EC, but relies on a gatekeeping role for primary care, which is not possible when most patients self-refer.

After the implementation of Vula, there was a statistically significant decrease in the number of non-urgent ‘green’ patients. Although it is impossible to prove that Vula alone was responsible for this, it makes sense that deferring patients who were deemed not to require transfer to the district hospital decreased the number of ‘green’ patients in the EC. One could also imagine that if there were more referrals made by Vula, this impact would be even greater. Other specialities have also demonstrated a substantial decrease in unnecessary transfers when Vula was introduced.^21^

Nevertheless, one can extrapolate that at the ERDH EC, there is a problem with the route that patients take when seeking healthcare. Instead of visiting their PCF, they go straight to the district level hospital. Only one of the WCG facilities was open 24-h a day, 7-days a week, and access to primary care was, therefore, limited afterhours and on weekends. This issue may be addressed in the implementation of National Health Insurance (NHI) in South Africa as everyone will be obligated to register with a PCP and to follow the designated referral pathways and gatekeeping if they want to be covered by insurance.^28^

Coordination of care between primary care and district hospitals has been evaluated as poor for both in-patient and out-patient referrals.^9,28^ Using electronic health records can improve care coordination,^29,30^ although this is not yet a reality in South Africa. Vula improved coordination during the referral and whilst awaiting transfer by: ensuring clear and standardised transfer of information, supplementing text with pictures, enabling feedback and interaction to clarify or add clinical information or to report or advise on clinical management prior to transfer. Vula also appeared to improve relationships between doctors at different levels of the health system, with more trust and respect, as well as greater understanding of each other’s contexts. It was also easier to contact the user directly because of the cell phone numbers being available, and avoided time being wasted whilst using the hospital switchboards, which has been a major source of irritation and a barrier to communication between providers.^31^ Vula uses asynchronous communication, which is more convenient to use than synchronous communication, however it may lead to a delay of patient transfer if either user does not respond promptly.^32^ Vula, therefore, is effective at improving the coordination of care when patients move from primary care to the district hospital.

Healthcare providers desire feedback about the patients they refer.^4^ As shown in the data, there was very little feedback about the patient after transfer, this was despite the accepting doctors wanting to give feedback and the referring doctors wanting to receive it. Contextual factors seem to be largely responsible for this, even though the capability and motivation seem to be in place. Such factors include delays in transfer whilst awaiting the ambulance, patients being seen by different doctors on arrival as well as workload pressures. Patients who are admitted to the hospital will eventually be discharged with an electronic discharge summary that is accessible to primary care providers. However, there is no electronic system to provide information on patients who are assessed, managed and returned home for follow-up in primary care directly from the EC. In this study, although the Vula app had the potential to provide such feedback, it was not used for this purpose. This is a significant gap in coordination of care which will impact on future primary care.

Feedback can be formative to assist with capacity building or merely to inform the referring doctors of their patient’s outcome. Feedback received in a clinical setting about a specific case and given in a timely manner promotes adult experiential learning.^34,35^ This kind of learning may be more effective than traditional continuing professional development activities.^36^ The current referral system and work environment do not promote feedback to referring doctors and any feedback given is seen as ‘extra work’. If the current healthcare system allowed for feedback to be integrated into the patient’s care, a work environment would be created for PCPs that promotes learning. In some cases, the referring doctor was more experienced than the receiving doctor and learning could also occur through feedback to the receiving doctor. Currently, the potential for Vula to assist with professional development is not fulfilled.

Utilisation of Vula was inconsistent. Whilst all the doctors working in the WCG health centres were using the Vula app, none of the nurses were. Nurses tend to defer to doctors when a patient needs referral, but doctors are not always available.^29^ Only some GPs were using Vula to refer patients. It is clear that the nurse-driven COCT clinics were not using Vula. Although international evidence shows that nurses are less prepared to adopt new information technology,^37^ the nurses interviewed in this study were not resistant to using Vula. There were other factors involved that prevented its use such as nurses feeling the need for training, not receiving a formal invite to use it, needing to pay for their own data and feeling that there would be too much information asked of them by the receiving doctor. Unfortunately, it is not known why so few GPs were using Vula. Most GPs currently refer patients with a brief letter and without discussing them. This may meet the GPs’ needs for an efficient disposal of the patient at the end of the consultation, but does not do justice to the need for better coordination of care and appropriate referrals.

Data from the Vula online dashboard showed some unexpected results. The age distribution of patients was relatively young. The life expectancy of the population in the catchment area of ERDH was relatively low with few elderly people.^22^ The diagnoses noted to account for the majority of the referrals over Vula may be common in a younger population, such as psychosis because of substance abuse. The burden of diseases in South Africa compared to the data obtained in the study are not the same.^38^ The lack of trauma from the data is because of ERDH not being a trauma centre, so very few trauma patients were referred. There was also no obstetric service, which accounts for few pregnancy complications. Referrals were recorded with the primary diagnosis and not with the other underlying causes. For example, pneumonia, accounting for 5.5% of the referrals, did not differentiate between bacterial pneumonia or TB; and anaemia, accounting for 4.8% of the referrals; may have been due to chronic renal failure from hypertension or diabetes, or chronic diseases like HIV and TB.

Limitations of the study include:

- Data collected from the EC triage logbook relied on accurate information being recorded. Not all the logbooks for selected dates could be traced at the time of data collection. This required the random selection process to be repeated and different months used for the data collection. The new time frame used may not fully account for seasonal variability, but is unlikely to affect the main results of the study. The triage process was not always accurate as the allocated colour relied on the accuracy of vital signs taken by the triage nurse.
- It is possible that additional perspectives might have been obtained by interviewing staff from the COCT clinics and GPs, who were not using Vula.
- Prior to using Vula, the amount of feedback is thought to have been very little. Unfortunately, it was not possible to collect data on the amount of feedback given before Vula was introduced and therefore a direct comparison cannot be made. The qualitative data suggests an increase in the amount of feedback and interaction.
- For the purposes of the study, patients triaged as ‘green’ were deemed inappropriate for a district level emergency centre. This is not always the case as the triage process has some flexibility in assigning the colours and at times can either underestimate or overestimate the triage colour. In general, however, patients triaged as green are regarded as non-urgent and encouraged to attend their PCF.

Eerste River District Hospital is a small district hospital in the metro health services and the findings would most likely be similar in the other small district hospitals that are organised and staffed in the same way, and which serve similar populations and PCFs. Findings from larger district or regional hospitals, with specialist departments managing their own referrals, might well be different.

The implications of the findings are to:

- Encourage increased utilisation of Vula to improve adherence to the continuum of care pathways and coordination between primary care and district hospitals. This should include use by nurses in all government facilities and private GPs.
- Encourage receiving doctors to provide feedback on the disposition of patients in the EC, particularly when they are discharged back home without admission in order to improve coordination of care between district hospitals and primary care.
- Encourage doctors using Vula to be more interactive in their conversations when referring and accepting patients to create an ongoing learning environment.
- Attention should be given to reducing the number of people who self-refer to the ECby, for example, improving access to primary care and use of gatekeeping.^39^

## Conclusion

The Vula Mobile app was used by all doctors working in WCG PCFs, and a few private GPs, but only accounted for a minority of patients in the EC. Vula improved coordination of care from primary care to the district hospital, but not vice versa. Vula had the potential to support continuing professional development and learning, but this potential was also largely missed. Utilisation of Vula app should be increased, particularly amongst nurses and private GPs, furthermore its potential to provide feedback should also be encouraged.
